# Innovation Model of China's High-End Equipment Industry: Do Social Capital and Dynamic Capabilities Matter for the COVID-19 Crisis?

**DOI:** 10.3389/fpubh.2021.683120

**Published:** 2021-06-07

**Authors:** Yuhong Ai, Diyun Peng

**Affiliations:** ^1^School of Management, Nanchang University, Nanchang, China; ^2^School of Economics and Management, Nanchang Hangkong University, Nanchang, China; ^3^School of Economics and Management, Nanchang University, Nanchang, China

**Keywords:** the COVID-19 crisis, social capital, dynamic capabilities, innovation model, qualitative comparative analysis

## Abstract

This paper explores the different model combinations of enterprise innovation in China based on the roles of social capital and dynamic capabilities. We implement Qualitative Comparative Analysis to understand the non-linear asymmetric relationships better. We use the data of 44 Listed Companies in China's high-end equipment manufacturing industry and find that three innovation models (the market-oriented independent innovation, government-supported technological innovation and industry-supported learning innovation models) are valid. Social capital, dynamic capabilities, and intra-industry networks are the main determinants of these innovation models. We also discuss the implications of these innovation dynamics on Chinese enterprises as a way to sustain the economy's high-quality development, including during the era of the COVID-19 pandemic crisis.

## Introduction

Innovation is becoming an important engine for China's economic growth and social development and is a strong driving force for economic transformation and upgrading. Since the introduction of the innovation-driven development strategy in 2012, there have been remarkable achievements in innovation and development in China, which ranks 14th in the Global Innovation Index. The quantity of researchers, patent citations, and scientific and technological publications is in first place. Although the number of innovations in China has increased substantially, there is still a marked difference compared with other countries like the United States (US), European countries, Japan, South Korea, in terms of quality and innovation, the conversion rate of scientific and technological innovation achievements. Therefore, it is important to study the innovation drivers to improve the innovative willingness and innovation performance. Scholars also argue that social capital an important aspect of the social networks of an individual or organization that can mobilize existing and potential resources or the capability to obtain resources for survival and development, which are important non-market factors (1–3). The proper use of social capital plays an important role in overcoming environmental uncertainty, enhancing competitiveness, promoting new product development and technological innovation, and improving innovation performance (4–6).

However, possessing rich natural resources and social capital does not guarantee good innovation in business practice. The dynamic capability is then considered the key factor, influencing the core competency and improving the innovation performance, which is considered the capability to integrate, establish, and reconstruct internal and external competitiveness in response to environmental change (7). On the other hand, several scholars indicate that enterprises, which obtain a competitive advantage directly, improve enterprise performance through capability. However, some researchers verify that different dynamic capability dimensions play a partial or complete moderating role between and the social capital and performance of an enterprise. The social capital of enterprises positively impacts performance by the mediation of accumulation and dynamic upgrading capability (8, 9).

Scholars empirically analyzed the relationship between social capital, dynamic capability, and innovation performance based on regression methods, by paying attention to the impact of some factors on each other. The relationship between variables could be a non-linear relationship under multiple conditions; even the synergy between the variables is neglected by extant literature. Considering the non-linear relationship, we introduce the qualitative comparative analysis method, Qualitative Comparative Analysis (QCA), into the research to explore the innovation model of enterprises with different combinations of social capital, dynamic capabilities, and innovation performance. At this stage, this research is the first paper to use the QCA method for understanding the non-linear asymmetric relationship to the best of our knowledge.

The rest of the paper is organized as follows. Section Literature Review reviews previous papers on social capital and innovation performance. Section Data and Methodology explains the methodology. Section Empirical Findings discusses the empirical findings. Section Conclusions provides the conclusions.

## Literature Review

### Social Capital and Innovation Performance

Bourdieu (10) first proposed the concept of social capital from a resource-based view. Social capital is formally defined as a collection of actual or potential resources and is closely related to institutionalized social networks, the definition of which covers the sum of the organization and individual social capital. Nahapiet and Ghoshal (11) were the first scholars to put forward corporate social capital. The authors concluded that corporate social capital facilitates new intellectual capital based on structural, cognitive, and relational dimensions. The *Capability School* approach suggests that social capital is a kind of capability, which is the dynamic process of enterprises and individuals obtaining the necessary development resources by constructing a network of relationships (12, 13).

Research literature on the relationship between social capital and innovation performance in China is also abundant. For instance, Zhang (14) divided the social capital of enterprises into horizontal relational capital, vertical relational capital, and social-relational capital and empirically analyzed external social capital's role in improving technological innovation performance. Dai and Zhu (15) found out that social capital has a significant positive impact on an enterprises' innovation performance, while absorptive capacity moderates the relationship between social capital and innovation performance. Wang and Yang (16) divide social capital into the internal and external capitals of enterprises according to the relational dimension, the structural dimension, and the cognitive dimension. Their empirical results show that external social capital, based on cognitive trust and common language, is conducive to knowledge recognition; external social interaction with common institutions, such as the external social capital dimension. These issues help to acquire knowledge and promote the innovation performance of enterprises through knowledge sharing and application. Internal social capital also contributes to knowledge sharing and application, thereby contributing to its innovation performance. Xiong and Sun (17) show that relation intensity, trust, and sharing goals have a significant positive impact on implicit technical knowledge, while implicit technical knowledge acquisition is positively related to product innovation performance; the effect of social capital on the acquisition of explicit technical knowledge is different.

Meanwhile, explicit technical knowledge on product innovation performance is not obvious, which is recommended to improve relation intensity, trust, and shared goals to promote the performance of enterprise production innovation. Zhu and Wang (18) studied the impact on innovation performance from the perspective of horizontal social capital, vertical social capital, and oblique social capital. In this view, the mediating role of absorptive capacity can strengthen the positive influence of horizontal and vertical social capital on innovation performance. Oblique social capital is also transformed from a U-shaped relationship with innovation performance to a positive correlation because of its mediating role.

By reviewing social capital, we observe that an enterprises' social capital directly or indirectly influences innovation performance. There is a direct or an indirect positive relationship between the social capital dimension and innovation performance. In short, it is suggested that social capital has a significant impact on innovation performance.

### Dynamic Capabilities and Innovation Performance

The concept of dynamic capability is the extension of the resource-based view for adapting to rapidly changing environments (18, 19). The firm's resources include tangible and intangible assets where “intangible assets are the ultimate source of sustainable value creation” (20). It is intangible assets such as capacity that can create unique competitive advantages over competitors. Early theory of industrial organization, emphasizes the importance of the external environment, however, simultaneously, the dynamic capability view broke through the limitations of passive adaptation, and looks inside the organization, introducing initiative into strategic organization theory.

Scholars deconstruct the connotation and composition of dynamic capability from a variety of perspectives. Teece et al. (21) consider the dynamic ability to perform various functions and strategic activities. Wang and Ahmed (22) treat dynamic capacities as third-order competencies and an indication of ultimate organizational capability. Resources are identified as the zero-order capability and core capability, respectively, as first-order and second-order in the hierarchy of organizational capabilities. Dynamic capability is of great significance because special skills are needed for successfully transferring this ability (23).

Helfat et al. (23) define dynamic capacity as “the capacity of an organization to create, extend, or modify its resource base purposefully.” Güttel and Konlechner (24) explain that dynamic capacity is “the adoption of a firms' resource and capability base in rapidly changing environments.” Teece (19) further analyzes dynamic capabilities, outlining that they consist of perceiving opportunity, grasping opportunity, and creating opportunities. Eisenhardt and Martin (25) identified an enterprise's dynamic ability as the process of enterprise integration, acquisition, reconstruction, and the release of resources.

Possessing a variety of resources does not mean building and reconfiguring a resource base according to environmental change, which causes different performance. Many scholars take dynamic capability as a dependent and an intermediate variable. Teece (26) suggest that dynamic capabilities can directly lead to competitive advantage and improve performance in response to rapid environmental changes. Makkone et al. (27) conducted empirical research on companies in the maritime, media, food processing, and other industries. The authors found that dynamic capabilities can increase the proportion of new product sales, reflecting their positive impact on innovation performance. Su and Liu (28) constructed a mechanic model of innovation performance from three dynamic capability dimensions: market perception capability, multi-organization collaborative control capability, and organizational learning absorptive capability. The dynamic capability has a significant effect on product innovation performance through innovation strategy. Wu (29) separates dynamic capability into two dimensions: opportunity recognition and opportunity utilization, and empirically tests the mechanism on innovation performance, which concluded that opportunity utilization positively influences innovation performance, while opportunity utilization partially mediates the relationship between opportunity identification and innovation performance. Sun and Zhang (30) explore the impact of dynamic enterprise capability on innovation performance under the context of internationalization. The results show that dynamic capability can significantly improve innovation performance.

By reviewing dynamic capability research, various arguments about the specific mechanism of dynamic capability on innovation performance dominate the literature. Most scholars find that dynamic capability has a positive effect on the maintenance of competitive advantage. It is conducive to breaking path dependence, causing change, renewal, and enables the redeployment of the resource base in response to environmental changes to improve the innovation performance.

Based on the previous literature, it is clear that social capital and dynamic capacity are of great significance to innovation performance. Therefore, successfully managing social capital and dynamic capacity in different ways is crucial to achieving innovation performance. With this in mind, this study aims to empirically examine a successful innovation model in China's equipment manufacturing industries based on the QCA approach. Following our paper's empirical findings, firms can improve innovation performance by establishing their capital and capacities models, especially for the COVID-19 crisis era.

## Data and Methodology

### Sample and Data Collection

By accessing the information available from the China Stock Market and Accounting Research (CSMAR) Database, State Intellectual Property Office, and Wanfang Patent Database, we obtained information on 106 listed companies that met the basic criteria. After excluding companies with incomplete data, loss of profits, and ST shares, we selected 44 samples over the period from 2014 to 2018, with a total of 1,320 observations for six variables. The mean of the indices was finally selected spanning 5 years of sample data, aggregately 264 observations.

Table 1 shows the industrial range, operating age, and scale of 44 companies. Samples from intelligent equipment manufacturing account for more than 50%, followed by companies for marine engineering, and finally, one company for satellite manufacturing. All the companies have been in operation for a minimum of 10 years, 61.36% of which have more than 2,000 employees.

**Table 1:** Descriptive statistics of sample data.

**Industry**	**Number**	**Percentage (%)**	**Items**	**Category**	**Number**	**Percentage (%)**
Aviation Equipment	3	6.82	Operating age (year)	10–15	7	15.91
Satellite Manufacturing and Application	1	2.27		16–20	18	40.91
Rail Transit Equipment Manufacturing	6	13.64		>20	19	43.18
				Total	44	100
Marine Engineering Equipment Manufacturing	7	15.91	Scale (employee)	2,000 ≤	17	38.64
Intelligent Equipment Manufacturing	25	56.81		2,000–5,000	14	31.82
Photovoltaic Industry	2	4.55		>5,000	13	29.54
Total	44	100		Total	44	100

### Variables and Measures

#### Social Capital

Adler and Kwon (31) classify social capital into two categories: internal social capital, and external social capital. As discussed by Ma and Li (32) and based on the available data, entrepreneurship embodies political social capital, and business social capital, with ties to industry.

Political social capital was measured by the number of senior executives or government officials or the current or former deputies to the National People's Congress or the Chinese People's Political Consultative Conference (CPPCC). Executives in compliance with political identity were assigned 1 point, otherwise, they received 0. Only the highest administrative level for one person was counted. The aggregate value is the sum of people number with the company's specified identity (32). Government and enterprise relation is used to present political capital in the empirical finding.

Ties with industry were measured by the number of senior executives who joined relevant trade associations. Referring to Zhou and Lin (33), senior executives associated with industry and commerce or various trade associations were assigned 1 point, otherwise, they received 0. It is viewed as industrial relation in the empirical analysis.

#### Dynamic Capabilities

Teece et al. (7) identify dynamic capability as having the following dimensions: coordination and integration ability, learning ability, and reconfiguration ability. Referring to Sheng and Jiang (34), coordination and integration ability was measured by the ratio of total asset turnover; according to Zhao et al. (35), learning ability and reconfiguration ability are, respectively, estimated by the proportion of employees with a bachelor degree or above, and the return of assets.

#### Innovation Performance

This paper's innovation performance was measured by the number of applied patents in nearly 5 years. Technology innovation performance is of great significance to the equipment manufacturing industry. Simultaneously, to better measure the actual innovation ability, we distinguished utility model patents from applied patents and used the number of invention patents to measure the actual innovation ability. We tried to encourage deep insights into the different models, successfully achieving a measure of innovation performance by combining social capital with dynamic capability in the high-end equipment manufacturing industry.

This paper aimed to study the optimal combination of social capital and dynamic capabilities by comparing the innovation performance of different enterprises. The traditional statistical method was based on large sample data and a stochastic model that verified a causal relationship between a relatively small number of variables. However, according to the literature, innovation performance, dynamic capability, and social capital have non-linear asymmetric relationships, meaning the QCA is used as a research tool and is concerned with cross-case concurrency causality. Through configuration analysis, we can effectively solve the asymmetric causality problem. This method identifies the pre-factor configuration with the most explanatory power according to “consistency” and “coverage” parameters (36). The QCA technology is a case-oriented approach and suitable for small sample study, without being affected by the number of research samples.

## Empirical Findings

Sample observations are assigned 0 or 1 according to the relevant threshold. According to the observation above, the average level is given the value of 1, and a value of 0 is assigned to the observation below the average. The related results are shown in Table 2.

**Table 2:** Pre-due feature selection and assignment.

**Factors**	**Pre-factors**	**Measurement standards**	**Assignment**
Dynamic capabilities	Coordination and integration capability	Value of “coordination and integration capability” is greater than or equal to the sample median	1
		Value of “coordination and integration capability” is less than the sample median	0
	Learning capability	Value of “learning capability” value is greater than or equal to the sample median	1
		Value of “learning capability” value is less than the sample median	0
	Organizational reconstruction capability	Value of “organization reconstruction capability” is greater than or equal to the sample median	1
		Value of “organizational reconstruction capability” is less than the sample median	0
Social capital	Government-enterprise ties relations	Value of “government-enterprise relations” is above or equal to the sample median	1
		Value of “government-enterprise relations” is less than the sample median	0
	Industrial relations	Value of “industrial relation” is above or equal to the sample median	1
		Value of “Indusrial relation” is less than the sample median	0

Variables such as dynamic capabilities, social capital, and invention patents were converted into truth tables based on assignments (see Table 2).

In Table 3, the original consistency threshold was 0.9, so a configuration with 0.9 or higher consistency was set to 1 in the survival column and 0 for cases where consistency was lower than 0.9. “Standard analysis” was selected, then the pre-factor configuration was identified, the software presents complex solutions, straightforward solutions, and intermediate solutions. While presenting simultaneously simple solutions and intermediate solutions, the pre-factors are the core conditions (expressed by “U” and “~”). Existing in the intermediate solution but not in the simple solution, the pre-factors are the edge conditions (expressed by “U” and “.”). The pre-factor configuration for the innovation output is shown in Table 3.

**Table 3:** Truth table.

**Dynamic capabilities**			**Social capital**		**Quantity**	**Innovation output (PO)**
**Integration capability (IC)**	**Learning capability (LC)**	**Organizational capability (OC)**	**Government-enterprise relations (GR)**	**Industrial relations (IR)**		
1	0	1	0	0	4	0
1	0	1	1	0	3	1
1	0	0	1	1	3	0
1	0	0	0	0	2	0
1	1	1	1	0	2	1
1	1	1	0	0	1	1
1	1	0	1	0	1	1
1	0	0	0	1	1	1
1	0	1	0	1	1	0
1	1	1	0	1	1	1
1	0	1	1	1	1	1
1	1	1	1	1	1	1
0	0	0	0	0	5	0
0	0	1	1	1	3	0
0	1	0	0	0	2	0
0	1	0	1	0	2	0
0	0	1	1	0	2	0
0	1	0	0	1	2	1
0	1	1	0	1	2	1
0	0	0	1	1	2	0
0	0	1	0	0	1	0
0	1	0	1	1	1	1
0	1	1	1	1	1	1

As shown in Table 4, the five pre-factor configurations' consistency was above 0.9, indicating that all existing pre-factor combinations meet the consistency criteria' requirement and promote performance innovation output. The sign “.” and “~” indicate that the condition exists, and “U” represents that the condition does not exist.

**Table 4:** Configuration.

**Variable**	**C1**	**C2**	**C3**	**C4**	**C5**
IC	U	~	~	.	.
LC	~	~		.	U
OC		~	~		U
GR			~	.	U
IR	~			U	~
Consistency	1	1	1	1	1
Raw coverage	0.25	0.2083	0.2917	0.125	0.04167
Solution coverage	0.7083				
Solution consistency	1				

We combine all the configurations with the core conditions in the complex solution. It is concluded that the three configurations of IC-LC-OC, IC-OC-GR, and LC-IR have more explanatory power with a consistency of 1. The results are shown in Table 5. The three coverage of the configurations was 0.458, 0.292 and 0.25, respectively (see Table 5). According to the higher-order configuration of the core conditions, the combination of dynamic ability, social capital, and innovation output can be summarized into three models.

**Table 5:** Major configurations of innovation output.

**Variable**	**C1**	**C2**	**C3**
IC	~	~	
LC	~		~
OC	~	~	
GR		~	
IR			~
Consistency	1	1	1
Coverage	0.458	0.2917	0.25

The market-oriented independent innovation model, i.e., the IC-LC-OC configuration: The core condition of the configuration were the three elements of the integration and coordination capability, learning capability, and organizational transformation capability, which indicates that high-end equipment manufacturing industry enterprises are more willing to take the initiative if they possess a strong dynamic capability.

The first finding was that enterprises that can integrate and coordinate internal and external resources according to market demand and are more willing to innovate future strategies. Second, enterprises with excellent learning capability can also fully use inner and external knowledge to realize the diversification of explicit knowledge replication and tacit knowledge innovation. The more knowledge the enterprises master, the more conducive they are to fostering innovation. Third, enterprises with stronger organizational transformation ability often take the initiative to carry out technology innovation because they can adjust organizational strategy and development model according to environmental change. This model shows that enterprises with dynamic capability are more willing to adopt the “independent innovation model” to improve the performance of the high-end equipment manufacturing industry.

The government-supported technological innovation model, i.e., the IC-OC-GR configuration: The government-supported technological innovation model's core conditions include integrating and coordinating ability, organizational transforming capability, and the good relationship between government and enterprise. In this model, the government's social relations play a leading role, and the dynamic ability of enterprises takes a back seat. In technological innovation, high-end equipment manufacturing enterprises need a lot of capital and policy support. A good relationship with government agencies is therefore an important means for enterprises to gain a competitive advantage. On the one hand, good government-enterprise relations can increase the probability of obtaining competitive financial subsidies and useful policy information. They can re-integrate resources and even change the organizational structure to meet the requirements of national strategies. Under this circumstance, even without strong learning ability, the enterprise still desires to adopt technology-based innovation to achieve innovation targets.

The industry-supported learning innovation model, i.e., the LC-IR configuration: The configuration's core conditions consist of learning ability and good industrial relations. Industry associations can provide a development platform for equipment manufacturing enterprises with learning ability. It can provide enterprises with information on industry development and market demand and guide enterprises to carry out strategic reforms. It also provides strategic opportunities for enterprises to search the key knowledge of employees, perform technological innovation and achieve cooperation to promote the enterprise's willingness to choose innovation. Knowledge about marketing and distribution channels from industrial associations accelerates the transformation of technology innovation. This model stresses that industrial supports promote enterprise to choose innovation.

## Conclusions

Based on a sample of 44 listed companies in the high-end equipment manufacturing industry, this paper has studied the mechanism of dynamic capability and social capital on technological innovation performance. Findings indicate that market-oriented independent innovation and industry-supported learning innovation models are valid. The results show that the innovation model includes different combinations of dynamic capability and social capital. Equipment manufacturers with good dynamic capabilities tend to choose technology innovation. Dynamic capability means that equipment manufacturing enterprises can integrate and reset internal resources, respond to changes in the external environment, capture and master the outside world's key knowledge and technology by using independent learning ability and learning mechanisms to achieve competitive advantages. Dynamic capability and adaptation to the environment help organizations explore market opportunities and withstand market challenges. Therefore, enterprises take the initiative to engage in technological innovation.

Government support is an important condition for these enterprises to adopt technology-style innovation. As a capital-intensive, technology-intensive, and labor-intensive industry, substantial financial, intellectual, and labor factors are required for industrial development. Maintaining good interaction with government departments helps enterprises access important policy information and preferential policies in a timely manner, and even enables more opportunities to participate in the government's important construction projects, which provides tremendous help for equipment enterprises to cope with uncertainty in the market, effectively improving the willingness to carry out technology-oriented innovation activities. A good industrial platform encourages enterprises to choose a technology-style innovation model. Industry associations, industrial and commercial organizations, and other organizations can provide support during strategic development. The knowledge platform these trade associations offer is very important for enterprises in terms of their learning ability.

The suggested policy implications for the COVID-19 era are, firstly, that equipment manufacturing enterprises need to have a good dynamic capability, as this is an influential factor in actively choosing a model of technical innovation. In addition to external social capital, enterprises also need to enhance their operational capability, increase investment in research and development, improve the efficiency of asset operations and product profitability, and create flexible organizational practices to maximize dynamic capability and adapt to environmental changes. Secondly, the platform role of trade associations promotes equipment enterprises to carry out technological innovation. Completing and improving the functions of industry associations in attracting capital and talents, drawing wisdom, and broadening channels further promotes technological innovation by equipment enterprises. Thirdly, strengthening government guidance and support will encourage enterprises to adopt technological innovation and increase the innovation output. Because of China's current innovation capability and level, the government needs to carry out micro-planning and top-level design to develop the equipment manufacturing industry, guide, and support the innovation activities of enterprises. Building up a good policy environment, constructing intelligent science and technology platforms, implementing key projects in major areas, and promoting industry synergism, science, and research are good measures of enhancing willingness toward innovation. The limitations in this study are that single measures are chosen for each capability, which leads to simplification of the research conclusions and the dichotomy of these observations results ignores other configurations. Further research on the innovation model is needed. At this stage, export quality and export diversification indices can be potential drivers for innovation, as Can and Gozgor discuss (37).

## Data Availability Statement

Publicly available datasets were analyzed in this study. This data can be found here: https://us.gtadata.com/; https://english.cnipa.gov.cn/; http://www.wanfangdata.com/.

## Author Contributions

YA wrote the manuscript and data collection. DP wrote the manuscript and estimations. All authors contributed to the article and approved the submitted version.

## Conflict of Interest

The authors declare that the research was conducted in the absence of any commercial or financial relationships that could be construed as a potential conflict of interest.
